# Drosophila Glue: A Promising Model for Bioadhesion

**DOI:** 10.3390/insects13080734

**Published:** 2022-08-16

**Authors:** Manon Monier, Virginie Courtier-Orgogozo

**Affiliations:** CNRS, Institut Jacques Monod, Université Paris Cité, 75013 Paris, France

**Keywords:** bioadhesion, glue, Drosophila, fly, Sgs, biomimetism, evolution, salivary gland, glycoprotein, mucin

## Abstract

**Simple Summary:**

Before entering metamorphosis, the larvae of Drosophila flies expel a transparent glue from their mouth, which solidifies in contact with air within seconds and fixes the animal to a substrate (wood, leaves, fruits, stones, etc.) for several days until the adult emerges. This glue displays interesting adhesive properties, as it can adhere to various substrates with strengths similar to strongly adhesive commercial tapes. We review here the production, aspect, composition and role of this glue in the model organism *Drosophila melanogaster* and in other Drosophila species. The glue is made of several proteins, which have diversified rapidly during evolution. With the large diversity of substrates and environmental conditions where fly species undergo metamorphosis, Drosophila glue provides a large source of inspiration for the development of biomimetic adhesive materials. We propose several potential avenues of research for the future development of Drosophila-inspired adhesive materials.

**Abstract:**

The glue produced by Drosophila larvae to attach themselves to a substrate for several days and resist predation until the end of metamorphosis represents an attractive model to develop new adhesives for dry environments. The adhesive properties of this interesting material have been investigated recently, and it was found that it binds as well as strongly adhesive commercial tapes to various types of substrates. This glue hardens rapidly after excretion and is made of several proteins. In *D. melanogaster*, eight glue proteins have been identified: four are long glycosylated mucoproteins containing repeats rich in prolines, serines and threonines, and four others are shorter proteins rich in cysteines. This protein mix is produced by the salivary glands through a complex packaging process that is starting to be elucidated. Drosophila species have adapted to stick to various substrates in diverse environmental conditions and glue genes appear to evolve rapidly in terms of gene number, number of repeats and sequence of the repeat motifs. Interestingly, besides its adhesive properties, the glue may also have antimicrobial activities. We discuss future perspectives and avenues of research for the development of new bioadhesives mimicking Drosophila fly glue.

## 1. Introduction

Bioadhesives are materials naturally produced by living organisms that can stick two separate items together and resist their separation [1]. These materials present singular physicochemical properties that have gone through millions of years of evolution. They are of commercial interest because they are made of proteins and sugars and hence are safe for the human body and the environment. Dental, medical and industrial applications often require adhesion in wet environments, and the marine mussel’s byssus has become a leading model for biomimetic wet adhesion [2,3]. Still, mussel-inspired bioadhesives are so far only used in research and have not been tested through clinical trials [2]. They have been used as a model to perform sutureless wound closure or to seal a fetal membrane. These glue components may also have anticancer and antimicrobial applications thanks to their sticky properties at the cellular level that enable them to target cancer or microbe cells. They also possess antifouling properties that can be used to control absorption of cells or proteins into a surface [3].

In contrast, bioadhesives that work in dry environments are less well characterized. The glue produced by Drosophila flies to stick themselves to a substrate for several days during metamorphosis appears to be a promising model for biomimetic dry adhesion. This glue is produced by the animal at the third instar larval wandering stage [4], a developmental stage during which the Drosophila larva does not feed and is searching for an appropriate site to undergo metamorphosis [5]. The glue is secreted by exocrine cells and accumulates into a pair of salivary glands. Just before entering into metamorphosis, the larva expectorates the entire content of the glands within a minute and the glue is spread all over the body. The glue solidifies rapidly and forms a transparent dry material located at the interface between the substrate and the animal [4]. After expectoration of the fluid, the larval skin hardens and encloses the now-immobile animal. The process that includes the hardening of the larval skin and the adoption by the animal of a characteristic barrel-like shape is named pupariation [5]. At the end of pupariation, the animal is a prepupa. Between 4 and 6 h after pupariation, the epidermis comes off the puparium cuticle, and a gas bubble appears in the abdomen. Eight hours later, the animal molts and technically becomes a pupa [5]. A few days later, an adult emerges and moves out from the pupal case. The glue allows the animal (as a prepupa and then as a pupa) to remain attached for several days onto its substrate, despite temperature variation, wind, rain and other environmental factors. There has likely been strong evolutionary pressure for firm attachment, as it allows the animal to remain within the environment it chose for metamorphosis, thus increasing its chances of survival.

Given the wide diversity of environmental conditions in which they live and the variety of substrates to which they attach, the numerous fly species that produce glue represent a large source of inspiration for biomimetism. Just within the Drosophila genus, more than 1600 species have been described, and they are widely spread around the world [6], with some species present on all continents while others are specific to an island, tropics or deserts [7]. The glue being located at the interface between the animal and the pupariation substrate, its composition and properties might be adapted to the nature of the pupa’s microhabitat. Experiments in the laboratory have found that different species and strains choose distinct pupariation sites according to humidity, light, temperature, larval density, substrate texture and substrate consistency [5,8]. For example, in laboratory conditions, *D. busckii* and *D. simulans* prefer to pupariate on humid surfaces, while *D. melanogaster* and *D. hydei* prefer dry substrates [9]. *D. simulans*, *D. yakuba*, *D. mauritania* and *D. malerkotliana* are found to pupariate in fruits rather than on glass walls, whereas *D. melanogaster*, *D. ananassae*, *D. virilis*, *D. novamexicana* and *D. hydei* prefer to pupate on the vial walls [10]. Interestingly *D. carcinophila* and *D. endobranchia* have adapted to a humid environment as their pupae attach to the surface of the external mouthparts of land crabs [11,12]. Unfortunately, due to their small size (on the order of 1–2 mm length) and their brown color, which is usually hardly distinguishable from the environmental background, pupae are difficult to spot in nature and there is little information about pupariation sites in the wild for the various Drosophila species. While species with narrow ecological niches are expected to have precisely defined pupariation sites, others appear to stick to a large range of substrates. *D. melanogaster pupae* have been found adhered to multiple substrates, including the dry parts of various rotten fruits, grape stalks and wood [9,10,13,14]. *D. simulans* and *D. buzzatii* pupae have been observed on the dry parts of *Opuntia ficus-indica* cactus [15]. The invasive species *D. suzukii* and many Hawaiian Drosophila species often pupariate several centimeters deep in the soil [12,16,17]. The pupariation sites described in the literature might constitute only the most visible locations, while other pupa microhabitats, which are not easily accessible, may not be recorded. 

In the 1970s and 1980s, the proteins that make up the *Drosophila melanogaster* glue were characterized biochemically, and their corresponding *salivary gland secretory* (*Sgs*) genes were identified. The glue genes then became a premier model to study the regulation of gene expression, with several ecdysone pulses triggering their expression at defined developmental stages. Such studies were facilitated by the presence of polytene chromosomes in the salivary gland cells [18]. Polytene chromosomes are giant chromosomes visible with classical light microscopy that are made of hundreds of sister chromatids packed together, resulting from multiple rounds of endoreplication. A larva possesses one pair of salivary glands, with about 130 secretory cells per gland in *D. melanogaster* [19]. Each secretory cell contains about a thousand chromatids for each chromosome, thus allowing the production of large amounts of adhesive glue within a short amount of time [20].

Although the regulation of glue gene expression has been extensively studied, comparatively very little is known about the function and the adhesive properties of the glue, in *D. melanogaster* or in any other Drosophila species. We review here the aspect and ultrastructure of the glue, its adhesive properties, function, its composition in *D. melanogaster* and other Drosophila species and its potential for developing bioadhesives.

## 2. Research Interest in the Adhesive Properties of Drosophila Glue Is New

We searched for “Drosophila glue” in PubMed on 7th June 2022 and retrieved 152 research articles (Table S1). Among them, 32 were not relevant and 120 dealt with the glue produced by Drosophila larvae to attach the animal to a substrate during metamorphosis. We attributed to each article one of the following research topics: glue gene expression, glue gene identification, glue secretion, glue of other Drosophila species, salivary gland physiology and glue ultrastructure and adhesion.

**Figure 1:**
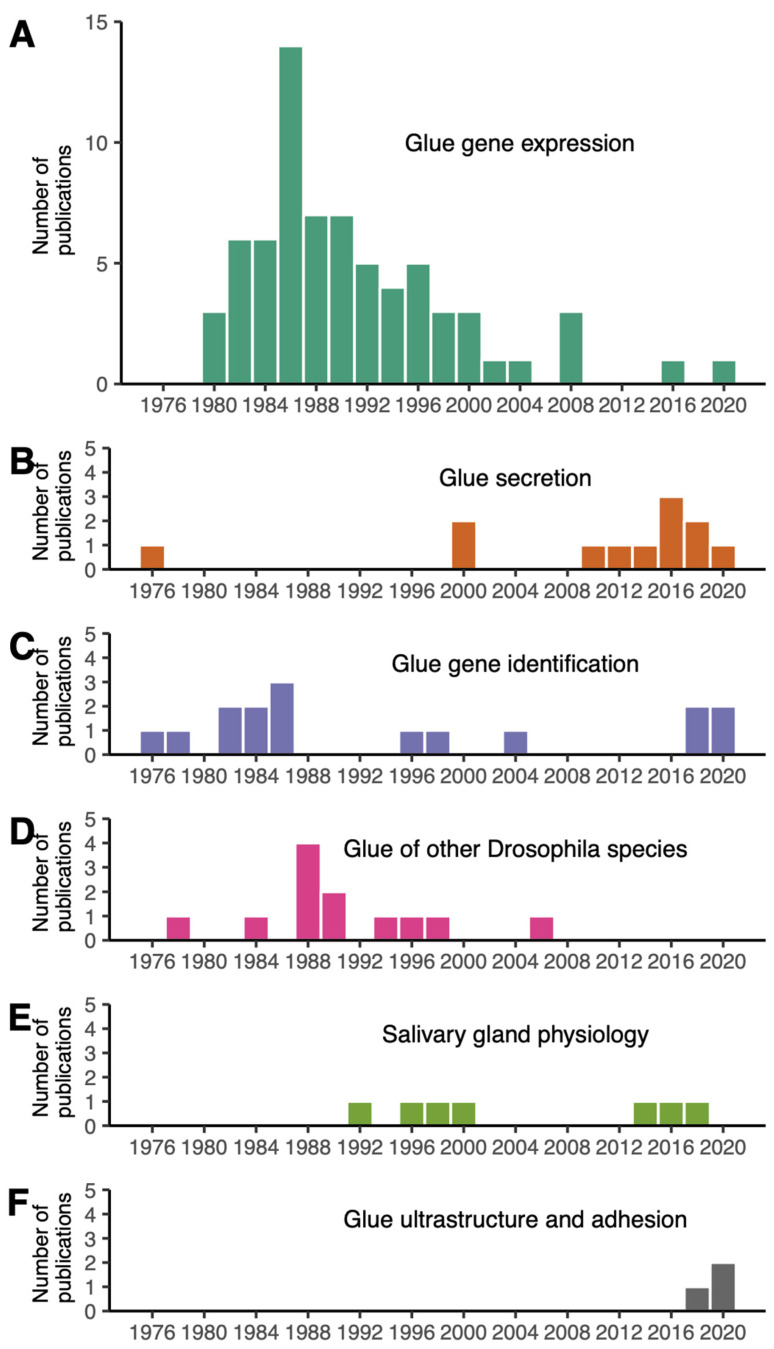
Distribution of Drosophila glue papers by year of publication and research topic. Five research topics are distinguished: (**A**) glue gene expression (70 articles), (**B**) glue gene identification (15 articles), (**C**) glue secretion (12 articles), (**D**) glue of other Drosophila species (12 articles), (**E**) salivary gland physiology (7 articles), (**F**) glue ultrastructure and adhesion (3 articles).

The earliest sets of papers, starting from 1975, focused on glue secretion and glue gene identification (Figure 1). Papers published before 1975 were not retrieved by this PubMed search because abstracts are not included in the PubMed database for most articles published before 1975 (https://pubmed.ncbi.nlm.nih.gov/help/, accessed on 8 June 2022). Note that this review article also includes older papers and publications not found with these keywords. 

More than half of the collected Drosophila glue papers were devoted to the regulation of glue gene expression, with a peak in publication number in the 1980s (Figure 1A, File S1). A few papers, classified as “salivary gland physiology”, examined diverse aspects of the salivary glands, including programmed cell death and movements of various ions and metabolites occurring after glue secretion at later stages during metamorphosis (Figure 1E). Although the role of the glue in fixing the animal to a substrate was proposed by G. Fraenkel and Victor J. Brookes in 1953 [4], research interests in the adhesive properties of this glue are fairly recent. Surprisingly, we found only three papers focusing on Drosophila glue ultrastructure and adhesive properties, published in 2019–2021 (Figure 1F).

## 3. Aspect and Ultrastructure of Drosophila Glue

**Figure 2:**
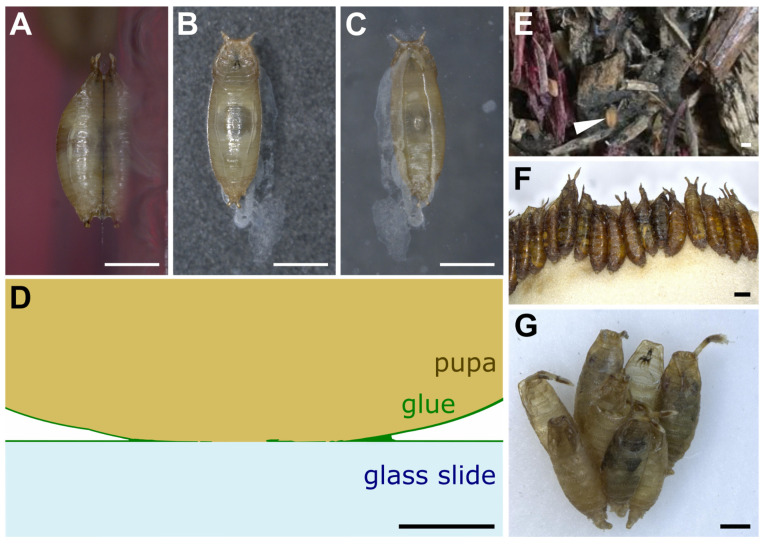
Drosophila pupae attached to various substrates. (**A**–**C**) *D. melanogaster* pupa attached with its own glue to a glass slide. Three pictures were taken of the same individual: (**A**) side view, (**B**) dorsal view, (**C**) ventral view throughout the glass slide. Anterior is up. White prints correspond to glue tracks on the glass slide or secretion from the larva before pupation. (**D**) Schematic transverse section of part of a *D. melanogaster* pupa attached to a glass slide with its own glue. Drawing based on confocal microscopy sections of *Sgs3:GFP* pupae obtained as in [21]. (**E**) First centimeters of soil made of wood chips from Bassevelle, France, where a Drosophila pupa (arrowhead) was found in July 2018. (**F**) Array of *D. hydei* pupae naturally attached to the plug within a laboratory vial. (**G**) Cluster of *D. acanthoptera* (Cornell University Drosophila Species Stock Center, stock #15090-1693.00) pupae found on the plastic wall of a laboratory vial. Scale bar is 1 mm in all panels except panel D, where it is 100 µm.

When left on glass slides, *D. melanogaster* animals usually attach on their ventral side, which presents a relatively flat surface, whereas the lateral and dorsal sides are more curved (Figure 2A–C). The glue forms an oval-shaped patch of solid transparent material of approximately 2 mm in length and 0.5 mm in width located at the surface of contact between the animal and the substrate (Figure 2C) [21]. Due to the overall barrel shape of the pupa, glue thickness varies from 0 μm (at the confocal microscope detection limit, in the middle of the surface of contact) to 20 μm (on the edges of the surface of contact). In addition, the glue can be detected when spread as a thin layer of about 0.1 μm onto the substrate outside of the surface of contact, and it also covers the surface of the pupal case that is not in contact with the substrate. It is thus reasonable to assume that the glue has good wetting properties both on the pupa and natural substrates, which probably means that the glue is highly hydrophilic. Further investigation on wetting properties and contact angle measurements will be valuable.

On the substrate near the posterior part of the animal is often found a whitish material that appears to be expelled by the intestine prior to metamorphosis and that mixes partly with the glue that is expectorated from the anterior part of the larva (Figure 2B,C) [21,22]. Whether this whitish material contributes to the adhesive properties of the glue is unknown.

In the wild and in the laboratory, pupae can be attached to the substrate either in isolation or in clusters (Figure 2D,E) [14,23,24]. When clustered, pupae are usually aligned with their anteroposterior axes pointing to the same direction (Figure 2D,E) [25]. In the wild, when pupating on Opuntia cactus, *D. buzzatii* and *D. simulans* tend to form species-specific aggregations in different locations on the cactus [15]. This clumping behavior, in which individuals of a given species closely group with each other, might improve the animals’ attachment, as their glue may combine with the glue of other already attached individuals. 

Scanning electron microscopy reveals that the surface of the glue is uniformly smooth and that its internal aspect is complex and structured [21,22]. The glue seems to be organized in thin layers separated by air bubbles [21] and is made of a multidirectional arrangement of thick fibers of various densities ranging from 30 to 90 nm diameter [22]. The variability in inner glue fiber thickness may serve as an elastic buffer that can accommodate mechanical stress exerted onto the animal and may allow its firm attachment to the substrate [22].

## 4. Adhesive Properties of the Glue

The adhesion force at the bonding surface of a bioadhesive can be measured in the laboratory as the force required to detach one material from the other under the application of a shearing, tensile or peeling force [1]. Recently, three studies of our group and collaborators used an automated pull-off adhesion test program to evaluate the force required to detach pupae from a substrate [21,26,27]. Third instar wandering larvae were let to pupariate on glass slides and kept in a box with wet paper. Fifteen to twenty-one hours later, pull-off force assays were conducted on pupae naturally attached to glass slides with their own glue using a universal test machine with a 5 N force sensor covered with double-sided tape. The glass slide with a pupa attached on it was placed under the force sensor. The force sensor was moved down until reaching the pupa, pressing onto it until a determined maximal force of 0.07 N, stilled at 0.03 N for 10 s and finally moved up at a constant speed of 0.2 mm·s^−1^ until a determined position. During the assay, three variables were measured: time (seconds), position of the force sensor (extension in mm) and force (N). The maximal value of the force reached during the assay when the pupa detaches from the glass slide was considered as the glue adhesion force for the individual.

The pull-off force for *D. melanogaster* pupae on glass slides was found to range from 151 mN to 269 mN with an average of 217 mN (15,500 times the weight of a pupa) [21]. By dividing the force value by the area of the pupa–substrate interface, which is approximately 1.1 mm^2^, the adhesive strength is thus estimated at 137–244 kPa (1 Pa = 1 N/m^2^) [21]. Adhesive strengths of the same order of magnitude (hundreds of kilopascals) are found for commercial adhesive tapes and for the mussel-inspired epoxy bioadhesives (136 kPa) [28]. In comparison, the cyanoacrylates composing highly adhesive glues, known as Super Glue, have a lap-shear force 100 times higher, of about 13.7 MPa [29].

Interestingly, diverse substrates, non-coated, Poly-L-lysine-coated (PLL-coated), poly-L-lysine–polyethylene glycol-coated (PLL–PEG- coated) and oxygen-activated glass slides present very similar adhesion forces ranging from 184 mN to 229 mN. In these cases, the break most often occurs between the pupal case and the glue, indicating that the bond between the glue and the substrate is stronger than the bond between the glue and the animal. This also suggests that the assay measures the adhesion force between the animal and its bioadhesive and not with its pupariation substrate. This observation can explain the similarity in adhesion forces between different substrates. Similarly, with substrates of increasing roughness, the break usually occurs between the pupal case and the glue, and no significant amelioration of the adhesion is detected [21]. As a control, when a low-stickiness substrate such as polytetrafluoroethylene (PTFE, Teflon) is used as a pupariation substrate, during the assay the glue completely detaches from the substrate and remains on the pulled pupa, and the average adhesion force is significantly lower (42 mN).

Taken together, these results mean that the bond between the glue and many different types of substrates is stronger than 200 mN, except for Teflon. No effect of humidity, temperature, atmospheric pressure and age of the pupa was found on the adhesion force measure [21], but the ranges in temperature and age of the pupa were small (respectively, 23.5–27.9 °C and 3.5–23 h), so it is possible that more extreme temperatures and older pupae exhibit differences in adhesion forces.

Relatively high variation in adhesion forces was detected between pupae for the same substrate, even within the same strain, ranging for example from 80 mN to 430 mN between *D. melanogaster* individuals for glass slides [27]. This variation cannot be solely attributed to the measurement error, as the universal test machine has an accuracy of ±0.5%. The adhesion assay described here measures the adherence of naturally attached pupae, and it is possible that factors that are not controlled in the experiment greatly influence adhesion, such as animal size, shape, weight, position of the pupa on the substrate or the amount of glue produced. Ideally, it would be good to develop adhesion assays on extracted glue. Unfortunately, there is currently no means to trigger glue expectoration. A recent study [22] that managed to collect glue monitored the larvae under a stereomicroscope until glue expectoration and required action within a few seconds before the glue solidified completely, which is a time-consuming approach.

Comparison of 12 *D. melanogaster* lines from different geographical regions revealed that adhesion can also vary between strains of the same species [27]. Besides *D. melanogaster*, glue adhesion strength has been reported in only three Drosophila species so far [26]. *D. simulans* detach at a similar force (median of 234.2 mN) [26] to *D. melanogaster* (median of 217 mN) [21]. *D. hydei* have the highest force (median of 482.6 mN) and *D. suzukii* the lowest (median of 78.7 mN). Furthermore, the adhesion force correlates with the glue contact area between the pupa and the substrate for these three species [26] but not for *D. melanogaster* [21].

Noticeably, most Drosophila researchers who manipulated pupae in vials know from experience that the glue displays an interesting reversible adhesiveness property. Pupae can be detached from the glass or plastic vial to which they stick by adding a small drop of water, waiting about one minute for the glue to swell in the water and then using a small paintbrush to gently detach the pupa. Such detached pupae can then be placed in another location within the vial. When dried, the glue will strongly adhere again to the tube.

In conclusion, assays have been developed recently to evaluate the force of detachment of naturally glued pupae. These assays will be very useful in future years to assess the range of adhesion forces across various fly species, diverse substrates and various environmental conditions.

## 5. Production and Expectoration of the Glue

The glue is made of water and several proteins named glue proteins [30]. The production of glue proteins in *D. melanogaster* begins during the second half of the third larval instar with their synthesis in the endoplasmic reticulum, where they are folded and then transported to the Golgi apparatus via the formation of Tango1-mediated rings that act as docking points between the endoplasmic reticulum and Golgi [31]. There, some of the glue proteins are glycosylated, and all the glue proteins are packaged into vesicles, also named granules. As they leave the trans Golgi network, these granules are about 1 μm in diameter, and they will fuse with each other to give large mature granules, about 3 to 8 μm in diameter [32]. Each salivary gland in *D. melanogaster* contains between 2500 and 3000 individual secretory granules [19]. In the granules, three main ultrastructural components are observed: a paracrystalline component made of electron-dense filament bundles, electron-lucent discs and a fine particulate or electron-opaque matrix [19,33]. The formation, composition and properties of these individual components is starting to be studied [33]. Progressively, glue proteins appear to be densely packed and dehydrated in large vesicles, in a process involving granule acidification, chloride ions, calcium ions and glycosylation [33]. 

Four to five hours prior to expectoration, a pulse of ecdysone triggers exocytosis, and granules release their content into the salivary gland lumen in an actomyosin-dependent process [34,35,36]. Once the secretion begins, the paracrystalline structure is lost and the lumen is filled with an amorph secretion [37,38]. A rise in pH and disappearance of calcium ions lead to the unfolding and hydration of the granule contents with water coming from the hemolymph, increasing the total volume [39,40]. At the end of the third instar larval stage, the salivary gland becomes bloated and full of glue.

When a larva finds an appropriate substrate for pupariation, it expectorates the glue, and the content of the lumen of the salivary gland is expelled through the mouth [5]. The process of glue expectoration has been described recently in exquisite detail based on movies of *D. melanogaster* larvae expressing *Sgs3:GFP* fluorescent glue [41]. At the end of the larval stage, the larva everts its pair of anterior spiracles, which are respiratory openings through which air will pass during metamorphosis, and it moves less and less. The animal also acquires a characteristic barrel shape through increasingly strong whole-body contractions and then enters a tetanic contraction phase where ventral anterior segments contract and slightly arch the anterior half of the larva for 17–70 s. Then, an anterior peristaltic wave propagates from segment T2 to A2 in approximately 3 s, further squeezing the anterior segments. A few milliseconds later, the glue is expelled from the lumen of the salivary gland to the exterior of the animal. While the glue is being released, a series of coordinated peristaltic movements propagate forwards and backwards, starting from segment A2, and lead to the spreading of the glue throughout the whole body. Furthermore, during expectoration, the animal usually moves forward about half of its length, reaching its final pupariation site, where it typically waves its anterior end left and right a few times. From the tetanus phase to the head waving, about 60–70 s have elapsed. Then, occasional whole-body contractions occur for about 50 min: they help remodel the puparium shape and lead to the formation of the operculum (the part of the pupal case that will be opened up by the adult fly when it emerges at the end of metamorphosis), and the cuticle starts to harden. The same suite of behavioral events accompanying glue expectoration was observed in *D. virilis* [41], which diverged from *D. melanogaster* about 45 million years ago [42]. After expectoration, the glue is liquid and it hardens in a few seconds, depending on air humidity, and becomes completely dry and solid after 3–5 min [4,22]. The movements of the larva during expectoration allow the glue to completely wet the body and increase contact with the animal surface topography by flowing into the folds and crevices of the cuticle, thus maximizing adhesiveness between the animal and the substrate. To our knowledge, the behavior of larvae that stick themselves to already attached pupae has not been described.

In summary, the stickiness of the pupae to their substrate results not only from the biochemical properties of the glue but also from the behavior of the larvae, including its body shape remodeling and its spreading of the glue via peristaltic movements. The glue proteins display remarkable properties, allowing them to be packed and dried into granules, fluidified in the gland lumen and then solidified in contact with air.

## 6. Identification of the Glue Genes in *D. melanogaster*

The glue of *D. melanogaster* was first isolated in 1948 from the salivary glands by placing the glands into an ethanol solution and then dissecting the solid plug of precipitated glue [43]. However, it was only in 1975 that the composition of the secretion was studied [44]. Using acid–urea gel electrophoresis, it was found that the glue separates into several bands, corresponding to different proteins. In *D. melanogaster*, bands were labeled from one to five according to their increasing electrophoretic mobility, thus from large to small size [44]. In *D. melanogaster*, eight glue genes were found in total (Table 1). The genes responsible for each protein band, named salivary gland secretion (*Sgs*) genes, were cytogenetically mapped based on polytene chromosomes and polymorphism between several *D. melanogaster* strains, with presence/absence of certain bands on glue gel electrophoresis correlating with the presence/absence of certain puffs on polytene chromosomes. Puffs are enlarged regions on polytene chromosomes that form swellings where active transcription takes place [45]. Then, in the 1980s, thanks to DNA cloning and restriction mapping, the glue genes were among the first developmentally regulated genes whose DNA gene sequence was identified [46]. *Sgs1*, *Sgs3*, *Sgs4* and *Sgs5* gene sequences were thus found at four distinct chromosomal locations (Table 1). Band 2 was considered as a contamination, and no further analysis of this band was performed [47]. *Sgs5bis*, *Sgs7* and *Sgs8* and *Eig71Ee* were found in later studies and were named without correlation to the electrophoretic mobility of their corresponding proteins (Table 1). Another glue gene, named *Sgs6*, has not been identified yet. The corresponding protein is present in only some *D. melanogaster* strains such as Canton S [48,49], and its nucleotide sequence located in region 71C3-4 is still unknown today [49,50]. *Eig71Ee* was first studied for its ecdysone-induced gene expression [51] and was later found to be expressed in the salivary glands at the late third instar larvae stage [52]. The Eig71Ee protein is O-glycosylated [53], contains internal repeats similar to Sgs3 and Sgs4 and is rich in cysteines (8%) like Sgs4 [52]. *Eig71Ee* is also expressed in the hemocytes and gut, where it is involved in immunity and clotting [53]. 

In total, the sequence of eight glue genes has been described in *D. melanogaster*. These eight genes are among the ten most highly expressed genes in salivary glands at the wandering third instar larval stage [27]. It is possible that *D. melanogaster* glue contains other proteins that have not been characterized yet. Highly expressed genes in wandering third instar salivary glands include genes involved in transcription and translation, as well as several small uncharacterized genes encoding for secreted peptides with the same tissue-specific, stage-specific gene expression as the glue genes [27]. These genes may encode for additional components of the glue. 

## 7. Characteristics and Functions of the Glue Proteins in *D. melanogaster*

The eight glue proteins identified in *D. melanogaster* present a signal peptide, so that the resulting proteins are all destined to the secretory pathway. We can distinguish two groups of glue proteins: Sgs1, Sgs3, Sgs4 and Eig71Ee are relatively long proteins containing multiple cysteines and amino acid repeats that are rich in prolines, serines and threonines, whereas Sgs5, Sgs5bis, Sgs7 and Sgs8 are relatively short proteins that do not have internal repeats and are rich in cysteines [19,50]. The relative amount of each protein within the glue is not known [19,48], and their respective roles in the various steps of glue production (granule maturation, hydration in the salivary gland lumen, lubrication during expectoration, glue cementing, glue adhesion) have so far mostly been inferred based on their amino acid sequence.

In the first group of proteins, repeats containing serines and threonines are subject to O-glycosylation and are characteristic of secreted mucins [19,50]. Mucins are highly glycosylated proteins present in animal mucus that protect the epithelia from physical damage or pathogens [57]. Glycosylation makes the molecules very hydrophilic, which enhances solubility, adhesion and is probably important for rehydration of the secreted content of the granules in the salivary gland lumen during glue production [30,40,58]. Sgs3 is O-glycosylated in the T-rich region and the PTTTK repetitive domain, and this glycosylation is in part accomplished by PGANT9A and PGANT9B enzymes [32]. The exact nature of the sugars covalently attached to the serines and threonines of the glue proteins has not been characterized. Computer predictions of protein structure reveal that the repeated regions are intrinsically disordered: they lack α-helices and β-sheets and do not have a fixed three-dimensional structure [19,50]. They are enriched in prolines like other intrinsically disordered regions [59], and they may form long threads [50]. The number of repeats and total protein length vary across *D. melanogaster* strains [55,60]. Overall, the long, disordered and highly glycosylated glue proteins of the first group may help to increase solubility at high concentrations, allow the rapid rehydration of the vesicles content after exocytosis in the salivary gland lumen, enhance fluidity of the mixture during expectoration and improve adhesive properties of the glue once released [19,33].

RNAi-mediated reduction of O-glycosylation leads to more tightly packed electron-dense fibers within the salivary gland granules, suggesting that adjacent fibers are repelled via their negatively charged sugars [33]. In RNAi loss-of-function mutants of *Sgs1* and *Sgs3*, the electron-lucent discs and the filament bundles are, respectively, gone. This shows that these glue proteins are involved in the intense packaging of molecules into the vesicles, and the authors propose that Sgs1 forms the disc structures while Sgs3 adopts a bundled filament structure. 

Glue proteins of the second group contain α-helices and β-sheets. They may be involved in the nucleation of the densification process in Golgi vesicles [19]. The multiple cysteines present in these glue proteins and in those of the first group can allow the formation of disulfide bridges intramolecularly to build up the three-dimensional structure of each protein and also between glue proteins, for example, by cysteine oxidation when the glue comes in contact with air, to create a complex fibrous macromolecular material [19,50]. 

Except *Sgs4* and *Eig71Ee*, all the glue genes are only expressed in the salivary glands and at the third instar larval stage [35,61,62], suggesting that their function is restricted to the making up of the glue. *Eig71Ee* is also involved in immunity and clotting in the hemocytes and the gut [53], while *Sgs4* is expressed in proventriculus and salivary glands from late second to late third instar larval stages [63], but its exact role in these tissues has not been characterized. The protein sequences of Sgs4 and Eig71Ee may thus also be subjected to other functional constraints.

In summary, according to their amino acid sequences, the eight glue proteins of *D. melanogaster* appear to display remarkable biochemical properties. Further work is needed to decipher the respective roles of the various molecular components of the glue in glue production, hardening, adhesion strength and adhesion reversibility.

## 8. Glue Genes and Proteins in other Drosophila Species

Besides *D. melanogaster*, fly glue has been mostly studied in *D. virilis*, and it appears to be composed of fewer proteins than in *D. melanogaster* (Table 2). Compared to the five bands present on electrophoresis gel in *D. melanogaster*, only three bands are found [64]. The first band protein is encoded by the gene *Lgp1* [64], an ortholog of *D. melanogaster Sgs3* also named Sgs3a in a more recent study [50]. In the *D. virilis* genome, it is adjacent to another glue gene, named *Lgp3* or *Sgs3b* [50,58]. *Sgs3a* and *Sgs3b* result from a recent duplication in the *D. virilis* lineage [50]. Lgp1/Sgs3a and Lgp3/Sgs3b are major components of the glue and together represent 90% of its content [65]. The remaining 10 % correspond to a weakly glycosylated 15-kDa protein named Lgp2 whose sequence was not characterized at the time [66]. By BLAST, only three glue genes were identified recently in the genome of *D. virilis* [50], *Sgs3a*, *Sgs3b and Sgs5bis. Sgs5bis* protein has no internal repeat and is expected to be 15.9 kDa, so we suggest that Lgp2 and Sgs5bis are the same.

Besides *D. melanogaster* and *D. virilis*, glue protein composition has been examined in *D. gibberosa* and in seven species from the *D. nasuta* group, which all diverged about 45 million years ago from *D. melanogaster* [42] (Table 2): *D. n. nasuta*, *D. n. albomicans*, *D. n. kepulauana*, *D. kohkoa*, *D. s. albostrigata*, *D. s. bilimbata* and *D. s. sulfurigaster*. Using gel electrophoresis, multiple protein bands were found, some of them being glycosylated [67], but the corresponding gene sequences were not characterized. The number of bands ranged from nine in *D. n. nasuta* up to seventeen in *D. gibberosa* (Table 2) [68]. Intraspecific polymorphism in the number of bands was observed in *D. nasuta nasuta* and *D. s. neonasuta* collected in the wild [69].

The level of glycosylation of the glue proteins appears to vary between Drosophila species. For example, *D. virilis* glue is rich in different sugars such as glucose, mannose and galactose [70], whereas the one from species of the *D. suzukii* subgroup, *D. suzukii*, *D. rajasekari* and *D. lucipennis*, contains no or low amounts of glycosylation [71].

In two recent studies, the glue genes from 22 Drosophila species spanning from the *D. melanogaster* subgroup to *D. virilis* and *D. mojavensis*, which diverged about 45 million years ago from *D. melanogaster* [42], were uncovered by BLAST based on sequence similarity with *D. melanogaster* glue genes [19,50]. Among them, *D. virilis* has the lowest number of glue genes (only three) while *D. santomea* and *D. yakuba* have the highest (nine in total) [50]. Interestingly, each species has at least one representative for each gene group: one encoding a long protein rich in cysteines, prolines, serines and threonines and containing repeats and one encoding a short protein rich in cysteines (Table 2).

## 9. Glue Gene Evolution

The various glue genes that have been identified in Drosophila species can be grouped into three gene families based on their sequence similarities: one composed of *Sgs5* and *Sgs5bis*, one with *Sgs4* and one with the remaining genes (*Sgs1*, *Sgs3*, *Sgs7*, *Sgs8* and *Eig71Ee*) [50]. Genes of the last group show C-terminal and N-terminal sequence similarities and have an intron at the same position and phase, with the codon disrupted by the intron encoding for an alanine or valine at position 10 [50,58].

*Sgs* sequences can differ in length between species. Overall, the glue genes with internal repeats (*Sgs1*, *Sgs3*, *Sgs4* and *Eig71Ee)* vary much more in length than the other glue genes, due to variation in the number of repeats and in the size of the repeated motif [50]. The number of repeats can vary rapidly. For example, *Sgs1* contains about 13 repeated motifs in *D. mauritiana* and 40 in *D. simulans* [50], which diverged some 300 000 years ago [42]. Furthermore, some genes can have internal repeats while their paralogs do not, suggesting that a glue gene devoid of repeats can acquire repeated sequences and/or that a glue gene can lose all of its repeats during evolution. For example, *Sgs5* does not appear to contain repeats in *D. melanogaster* but does in *D. simulans* [50]. In *D. melanogaster*, three glue genes are located at the same chromosomal location, and they share sequence identity, suggesting that they come from ancient duplications: one, *Sgs3*, contains repeats, whereas the other two, *Sgs7* and *Sgs8*, do not. This suggests that the presence/absence of repeats can change across evolution. As described above, the presence/absence of repeats is associated with two distinct glue functions: the multiple prolines, serines and threonines present in repeats appear to generate long glycosylated filaments, whereas the shorter proteins devoid of repeats may contribute to the scaffolding of the glue via disulfide bonds. Two types of functional glue proteins may thus be formed within the same gene family. 

Noticeably, the *Sgs1-3-7-8-Eig71Ee* gene family has experienced a higher rate of gene losses and gene duplications than other gene families present in the *Drosophila* genomes [50]. The rapid evolution of glue gene sequences, in terms of gene number, number of repeats and repeat motifs, may be related to the rapid adaptation of glue adhesiveness to various environmental conditions.

## 10. Role of Drosophila Glue in Natural Environments

Drosophila salivary glands form during embryogenesis, and it is unclear whether they produce digestive enzymes during early larval stages [74,75,76]. In any case, salivary glands appear to be dispensable during larval life since individuals carrying salivary glands as closed sacs devoid of ducts due to mutation in the *eye gone* gene survive until the pupal stage and then die as late pupae or adults [77]. During the prepupal stage, the salivary glands produce a massive secretion distinct from the glue into the peri-exuvial cavity that lies between the metamorphosing pupa and its pupal case [78]. This secretion contains immune-competent and defense-response proteins and acts as a protective barrier against microbial infections.

The main function of salivary glands at the end of the third instar larval stage is the production of a glue that will affix the animal to a substrate [4]. Pupal adhesion can have several functions. First, it can allow the organisms to remain in a favorable environment (in terms of temperature, humidity, background color, etc.), resisting mechanical forces (wind, rain, other animals) that may displace pupae into adverse surroundings. Second, it may help the adult to emerge from the pupal case, although this possibility has not been examined experimentally as far as we know. Third, it can protect the pupae from predation. Recent work from our group showed that in a forest near Paris, *D. simulans* pupae naturally attached to a substrate are taken away less frequently than manually detached pupae [26]. Furthermore, experiments in the laboratory showed that attached pupae are predated less efficiently by ants, which take more time to consume them onsite and are not able to carry them back to the nest [26].

The glue covers the surface of the animal (Figure 2D) for several days, until the adult emerges from the pupal case. So, it is possible that this material has other functions besides stickiness. Pupae are vulnerable not only to predation but also to parasitism [79], fungal or bacterial infection and desiccation. The decaying fruits, which represent a major pupariation site for many Drosophila species [8,10,14], are especially exposed to desiccation and are rich in fungi and bacteria. Besides its adhesive properties, it is thus possible that the glue may have other functions, none of which have been investigated so far, as far as we know. For example, it may repel predators and parasitoids or make the pupae undetectable to them. Del Pino et al. proposed that components of the salivary gland secretion may act as pheromones [23]. The glue may also act as a preservative and avoid fungal or bacterial infections. The glycoproteins that make up the glue belong to the mucin family, and mucins are known to have antimicrobial properties [39]. In particular, the glue protein Eig71Ee, also named gp150, which is present in hemolymph, lies in structures that entrap bacteria [53]. Scanning electron microscopy showed that yeast-like organisms and coliform bacteria can be found and efficiently trapped within the glue of *D. melanogaster* individuals raised in the laboratory [22].

Further work on the properties and function of Drosophila glue would be extremely useful to get a better idea of the possible applications of future biomimetic adhesives.

## 11. Perspectives for Future Applications

Bioadhesives inspired from nature may be compatible with the human body and biodegradable and thus offer attractive properties compared to synthetic ones. Furthermore, they may display antifouling or antimicrobial properties. Recent measurements of Drosophila glue adhesiveness showed that it is equivalent to strong commercial adhesive tapes [21]. Indeed, we noticed in our adhesion assays that commercial tapes with low adherence led to detachment of the pupa from the tape and not from its substrate. Research on Drosophila glue may help in the future to develop new bioadhesives for dry environments, on polarizable surfaces. 

However, several difficulties remain. First, the volume of glue produced by each larva is relatively small, making it difficult to study the physical and biochemical characteristics of this glue. Second, there is no available method to trigger glue expectoration from the larva. When larvae are manipulated with forceps right before glue expectoration, they can revert the pupariation process, retract their anterior spiracles and start moving again to find another pupariation site [5]. Third, Drosophila glue is produced through a complex granule maturation process, involving pH change, calcium ions and chloride ions [33]. Such a maturation process may be difficult to reproduce in vivo, unless large progress is made in organoid and organs-on-chips research [80,81]. Alternatively, small molecular elements of the glue such as modified amino acids may be found to be key to the adhesion process, and new adhesives may be created by synthesizing polymers containing such molecules. For example, the catecholic amino acid 3,4 dihydroxy phenylalanine (DOPA), which is abundant in mussel adhesive proteins, plays an essential role in strong underwater adhesion, and polyethylene glycol polymers grafted with DOPA are being developed as mussel-inspired tissue adhesives [2]. 

In future years, the powerful genetic tools of *D. melanogaster* will definitely facilitate the study of the roles of the different players in the formation of the glue and its adhesive properties: glue proteins, glycosylation, pH, calcium ions, chloride ions, etc. The diversity of glues produced by various Drosophila species and adapted to various environments represents a promising reservoir for bioinspiration.

## 12. Conclusions

Research on the biochemical and physical properties of Drosophila glue is just starting. This is an exciting emerging field where multiple avenues of research are available to learn more about the fascinating biophysical attributes of Drosophila glue, including adhesive properties and antimicrobial activities, as well as its elaborate biosynthesis and secretion. Furthermore, understanding the specificities of the diverse glues produced by Drosophila strains and species in relation to their environments will provide insight into the development of Drosophila-inspired adhesives.

## Figures and Tables

**Table 1:** List of the main glue genes of *D. melanogaster* and their characteristics. See FlyBase (http://flybase.org, accessed on 8 July 2022) for further information.

*Sgs* Gene Name	Band	Chromosome	Cytogenetic Map	Other Gene Names	Number of Amino Acids	Amino Acid Composition and Glycosylation State	Reference
*Sgs1*	1	2L	25B4	*CG3047*	1286	Presence of repeats PTTTTPR/STTTTSTSR. Rich in cysteines, prolines, serines and threonines. Glycosylated.	[54]
*Sgs3*	3	3L	68C11	*CG11720*	307	Presence of repeats KPTTT. Rich in cysteines, prolines, serines and threonines. Glycosylated.	[55]
*Sgs4*	4	X	3C11-12	*CG12181*	297	Presence of repeats. Rich in cysteines, prolines, serines and threonines. Glycosylated.	[46]
*Sgs5*	5	3R	90B3-8	*CG7596*	163	No repeat. Rich in cysteines, prolines and serines.	[56]
*Sgs5bis*	-	3R	90B5	*CG7587*	142	No repeat. Rich in cysteines and prolines.	[50]
*Sgs7*	-	3L	68C11	*CG18087*	74	No repeat. Rich in cysteines.	[47]
*Sgs8*	-	3L	68C11	*CG6132*	74	No repeat. Rich in cysteines.	[47]
*Eig71Ee*	-	3L	71E5	*CG7604* *VII I71–7* *gp150*	393	Presence of repeats CTCTESTT/(R/K)TNPT. Rich in cysteines, prolines, serines and threonines. Glycosylated.	[52]

**Table 2:** List of the glue genes and glue protein bands identified in Drosophila species. *Sgs* genes written in bold correspond to proteins with internal repeats rich in serines, threonines and prolines. * An updated genome assembly [72] shows that *D. suzukii* actually contains one *Sgs3* gene and only one copy of *Sgs7* (data not shown). Nd: not determined.

Species	Number of Bands	Glue Gene Sequences Identified	Reference
*D. simulans* *D. sechellia*	nd	***Sgs1**; **Sgs3**; **Sgs4**; Sgs5; Sgs7; Sgs8; **Eig71Ee***	[50]
*D. mauritiana*	nd	***Sgs1**; **Sgs3**; **Sgs4**; **Sgs5**; Sgs7; Eig71Ee*	[50]
*D. santomea* *D. yakuba*	nd	***Sgs1**; **Sgs3**; **Sgs4**; **Sgs5**; Sgs5bis; Sgs7; Sgs7bis Sgs8; **Eig71Ee***	[50]
*D. erecta*	nd	***Sgs3**; **Sgs4**; Sgs5bis; Sgs7; Sgs8; **Eig71Ee***	[50]
*D. eugracilis*	nd	***Sgs1**; **Sgs3**; **Sgs3bis**; **Sgs5**; Sgs5bis; Sgs7; Sgs8; **Eig71Ee***	[50]
*D.takahashii*	nd	***Sgs1**; **Sgs3**; **Sgs5**; Sgs5bis; Sgs7; Sgs8; **Eig71Ee***	[50]
D. suzukii *	nd	***Sgs1**; Sgs4; **Sgs5**; Sgs5bis;* 4 copies of *Sgs7; Sgs8; Eig71Ee*	[50]
*D. biarmipes*	nd	***Sgs1**; **Sgs3**; **Sgs3bis**; **Sgs5**; Sgs5bis; Sgs7; Sgs8; Eig71Ee*	[50]
*D.elegans*	nd	***Sgs1**; **Sgs3a**; **Sgs3b**; **Sgs3c**; **Sgs5***	[50]
*D. rhopaloa*	nd	***Sgs1**; **Sgs3a**; **Sgs3b**; **Sgs3c**; **Sgs3d**; **Sgs5***	[50]
*D. ficusphila*	nd	***Sgs1**; **Sgs3a**; **Sgs3b**; **Sgs3c**; **Sgs5**; Sgs5bis; **Eig71Ee***	[50]
*D. kikkawai* *D. ananassae*	nd	***Sgs3a**; **Sgs3b**; **Sgs5**; Sgs5bis*	[50]
*D. bipectinata*	nd	***Sgs3a**; **Sgs3b**; Sgs7; Sgs8; Sgs5; Sgs5bis; **Eig71Ee***	[50]
*D. pseudoobscura*	nd	***Sgs3a**; **Sgs3b**; **Sgs3c**; Sgs5bis*	[50]
*D. willistoni*	nd	***Sgs3a**; **Sgs3b**; Sgs7a; Sgs7b*	[50]
*D. virilis*	3	***Sgs3a*** (or *Lgp1*); ***Sgs3b*** (or *Lgp3*) *Sgs5bis* (or *Lgp2*)	[50]
*D. mojavensis*	nd	*Sgs4; Sgs5; Sgs7*	[19]
*D. persimilis*	nd	*Sgs5; Sgs7; Sgs8*	[19]
*D. n. nasuta*	9	nd	[68]
*D. n. albomicans*	10	nd	[68]
*D. n. kepulauana*	12	nd	[68]
*D. kohkoa*	10	nd	[68]
*D. s. albostrigata*	12	nd	[68]
*D. s. bilimbata*	14	nd	[68]
*D. s. sulfurigaster*	13	nd	[68]
*D. gibberosa*	17	nd	[73]

## Data Availability

Not applicable.

## Supplementary Materials

The following supporting information can be downloaded at: https://www.mdpi.com/article/10.3390/insects13080734/s1, Table S1. List of papers retrieved from PubMed using a search for “Drosophila glue”. File S1. R Script used to make Figure 1.

## Abbreviations

A2	Abdominal segment 2
DOPA	3,4 dihydroxy phenylalanine
*Sgs* gene	Salivary gland secretion gene
T2	Thoracic segment 2

## References

[1] Bianco-Peled H., Davidovich-Pinhas M. (2015). Bioadhesion and Biomimetics: From Nature to Applications.

[2] Pandey N., Soto-Garcia L.F., Liao J., Zimmern P., Nguyen K.T., Hong Y. (2020). Mussel-Inspired Bioadhesives in Healthcare: Design Parameters, Current Trends, and Future Perspectives. Biomater. Sci..

[3] Kord Forooshani P., Lee B.P. (2017). Recent Approaches in Designing Bioadhesive Materials Inspired by Mussel Adhesive Protein. J. Polym. Sci. Part Polym. Chem..

[4] Fraenkel G., Brookes V.J. (1953). The Process by which the Puparia of Many Species of Flies Become Fixed to a Substrate. Biol. Bull..

[5] Ashburner M. (2004). Drosophila: A Laboratory Handbook.

[6] O’Grady P.M., DeSalle R. (2018). Phylogeny of the Genus Drosophila. Genetics.

[7] Markow T., O’Grady P.M. (2005). Evolutionary Genetics of Reproductive Behavior in Drosophila: Connecting the Dots. Annu. Rev. Genet..

[8] Godoy-Herrera R., Cifuentes L., Díaz de Arcaya M.F., Fernández M., Fuentes M., Reyes I., Valderrama C. (1989). The Behaviour of Drosophila Melanogaster Larvae during Pupation. Anim. Behav..

[9] Godoy-Herrera R., Silva-Cuadra J.L. (1998). The Behavior of Sympatric Chilean Populations of Drosophila Larvae during Pupation. Genet. Mol. Biol..

[10] Vandal N.B., Siddalingamurthy G.S., Shivanna N. (2008). Larval Pupation Site Preference on Fruit in Different Species of Drosophila. Entomol. Res..

[11] Carson H.L. (1967). The Association between Drosophila Carcinophila Wheeler and Its Host, the Land Crab *Gecarcinus ruricola* (L.). Am. Midl. Nat..

[12] Carson H.L., Wheeler M.R. (1968). Drosophila Endobranchia, a New Drosophilid1 Associated with Land Crabs in the West Indies. Ann. Entomol. Soc. Am..

[13] Sokolowski M.B. (1985). Genetics and Ecology of Drosophila Melanogaster Larval Foraging and Pupation Behaviour. J. Insect Physiol..

[14] Beltramí M., Medina-Muñoz M.C., Arce D., Godoy-Herrera R. (2010). Drosophila Pupation Behavior in the Wild. Evol. Ecol..

[15] Beltramí M., Medina-Muñoz M.C., Pino F.D., Ferveur J.-F., Godoy-Herrera R. (2012). Chemical Cues Influence Pupation Behavior of Drosophila Simulans and Drosophila Buzzatii in Nature and in the Laboratory. PLoS ONE.

[16] Grossfield J. (1978). Non-Sexual Behavior of Drosophila.

[17] Woltz J.M., Lee J.C. (2017). Pupation Behavior and Larval and Pupal Biocontrol of Drosophila Suzukii in the Field. Biol. Control.

[18] Crosby M.A., Meyerowitz E.M. (1986). Drosophila Glue Gene Sgs-3: Sequences Required for Puffing and Transcriptional Regulation. Dev. Biol..

[19] Farkaš R., Cohen E., Moussian B. (2016). The Complex Secretions of the Salivary Glands of Drosophila Melanogaster, A Model System. Extracellular Composite Matrices in Arthropods.

[20] Meyerowitz E.M., Crosby M.A., Garfinkel M.D., Martin C.H., Mathers P.H., Vijay Raghavan K. (1985). The 68C Glue Puff of Drosophila. Cold Spring Harb. Symp. Quant. Biol..

[21] Borne F., Kovalev A., Gorb S., Courtier-Orgogozo V. (2020). The Glue Produced by *Drosophila melanogaster* for Pupa Adhesion Is Universal. J. Exp. Biol..

[22] Beňová-Liszeková D., Beňo M., Farkaš R. (2019). A Protocol for Processing the Delicate Larval and Prepupal Salivary Glands of Drosophila for Scanning Electron Microscopy. Microsc. Res. Tech..

[23] Del Pino F., Jara C., Pino L., Godoy-Herrera R. (2014). The Neuro-Ecology of Drosophila Pupation Behavior. PLoS ONE.

[24] Medina-Muñoz M.C., Godoy-Herrera R. (2005). Dispersal and Prepupation Behavior of Chilean Sympatric Drosophila Species That Breed in the Same Site in Nature. Behav. Ecol..

[25] Ringo J., Dowse H. (2012). Pupation Site Selection in Four Drosophilid Species: Aggregation and Contact. J. Insect Behav..

[26] Borne F., Prigent S.R., Molet M., Courtier-Orgogozo V. (2021). *Drosophila* Glue Protects from Predation. Proc. R. Soc. B Biol. Sci..

[27] Borne F., Kulathinal R.J., Courtier-Orgogozo V. (2021). Glue Genes Are Subjected to Diverse Selective Forces during Drosophila Development. Genome Biol. Evol..

[28] Du D., Chen X., Shi C., Zhang Z., Shi D., Kaneko D., Kaneko T., Hua Z. (2021). Mussel-Inspired Epoxy Bioadhesive with Enhanced Interfacial Interactions for Wound Repair. Acta Biomater..

[29] Ebnesajjad S., Landrock A.H. (2015). Characteristics of Adhesive Materials. Adhesives Technology Handbook.

[30] Kress H. (1974). Temporal Relationships between Leaving Food, Ecdysone Release, Mucoprotein Extrusion and Puparium Formation in Drosophila Virilis. J. Insect Physiol..

[31] Reynolds H.M., Zhang L., Tran D.T., Ten Hagen K.G. (2019). Tango1 Coordinates the Formation of Endoplasmic Reticulum/Golgi Docking Sites to Mediate Secretory Granule Formation. J. Biol. Chem..

[32] Ji S., Samara N.L., Revoredo L., Zhang L., Tran D.T., Muirhead K., Tabak L.A., Ten Hagen K.G. (2018). A Molecular Switch Orchestrates Enzyme Specificity and Secretory Granule Morphology. Nat. Commun..

[33] Syed Z.A., Zhang L., Tran D.T., Bleck C.K.E., Hagen K.G.T. (2022). Orchestrated Restructuring Events During Secretory Granule Maturation Mediate Intragranular Cargo Segregation. bioRxiv.

[34] Rousso T., Schejter E.D., Shilo B.-Z. (2016). Orchestrated Content Release from Drosophila Glue-Protein Vesicles by a Contractile Actomyosin Network. Nat. Cell Biol..

[35] Duan J., Zhao Y., Li H., Habernig L., Gordon M.D., Miao X., Engström Y., Büttner S. (2020). Bab2 Functions as an Ecdysone-Responsive Transcriptional Repressor during Drosophila Development. Cell Rep..

[36] Tran D.T., Masedunskas A., Weigert R., Ten Hagen K.G. (2015). Arp2/3-Mediated F-Actin Formation Controls Regulated Exocytosis in Vivo. Nat. Commun..

[37] von Gaudecker B. (1972). Der Strukturwandel der larvalen Speicheldrüse vonDrosophila melanogaster. Z. Für Zellforsch. Mikrosk. Anat..

[38] Farkaš R., Šuťáková G. (1998). Ultrastructural Changes of Drosophila Larval and Prepupal Salivary Glands Cultured in Vitro with Ecdysone. Vitro Cell. Dev. Biol.—Anim..

[39] Syed Z.A., Zhang L., Ten Hagen K.G. (2022). In Vivo Models of Mucin Biosynthesis and Function. Adv. Drug Deliv. Rev..

[40] Lane N.J., Carter Y.R., Ashburner M. (1972). Puffs and Salivary Gland Function: The Fine Structure of the Larval and Prepupal Salivary Glands OfDrosophila Melanogaster. Wilhelm Roux Arch. Für Entwickl. Org..

[41] Heredia F., Volonté Y., Pereirinha J., Fernandez-Acosta M., Casimiro A.P., Belém C.G., Viegas F., Tanaka K., Menezes J., Arana M. (2021). The Steroid-Hormone Ecdysone Coordinates Parallel Pupariation Neuromotor and Morphogenetic Subprograms Via Epider-Mis-To-Neuron Dilp8-Lgr3 Signal Induction. Nat. Commun..

[42] Kumar S., Stecher G., Suleski M., Hedges S.B. (2017). TimeTree: A Resource for Timelines, Timetrees, and Divergence Times. Mol. Biol. Evol..

[43] Kodani M. (1948). The Protein of the Salivary Gland Secretion in Drosophila. Proc. Natl. Acad. Sci. USA.

[44] Korge G. (1975). Chromosome Puff Activity and Protein Synthesis in Larval Salivary Glands of Drosophila Melanogaster. Proc. Natl. Acad. Sci. USA.

[45] Russell S., Ashburner M. (1996). Ecdysone-Regulated Chromosome Puffing in Drosophila Melanogaster. Metamorphosis.

[46] Muskavitch M.A., Hogness D.S. (1980). Molecular Analysis of a Gene in a Developmentally Regulated Puff of Drosophila Melanogaster. Proc. Natl. Acad. Sci. USA.

[47] Meyerowitz E.M., Bond M.W., Crowley T.E. (1983). The Structural Genes for Three Drosophila Glue Proteins Reside at a Single Polytene Chromosome Puff Locus. Mol. Cell Biol..

[48] Velissariou V., Ashburner M. (1981). Cytogenetic and Genetic Mapping of a Salivary Gland Secretion Protein in Drosophila Melanogaster. Chromosoma.

[49] Akam M.E., Roberts D.B., Richards G.P., Ashburner M. (1978). Drosophila: The Genetics of Two Major Larval Proteins. Cell.

[50] Da Lage J.-L., Thomas G.W.C., Bonneau M., Courtier-Orgogozo V. (2019). Evolution of Salivary Glue Genes in Drosophila Species. BMC Evol. Biol..

[51] Restifo L.L., Guild G.M. (1986). An Ecdysterone-Responsive Puff Site in Drosophila Contains a Cluster of Seven Differentially Regulated Genes. J. Mol. Biol..

[52] Wright L.G., Chen T., Thummel C.S., Guild G.M. (1996). Molecular Characterization of the 71E Late Puff in Drosophila Melanogaster Reveals a Family of Novel Genes. J. Mol. Biol..

[53] Korayem A.M., Fabbri M., Takahashi K., Scherfer C., Lindgren M., Schmidt O., Ueda R., Dushay M.S., Theopold U. (2004). A Drosophila Salivary Gland Mucin Is Also Expressed in Immune Tissues: Evidence for a Function in Coagulation and the Entrapment of Bacteria. Insect Biochem. Mol. Biol..

[54] Roth G.E., Wattler S., Bornschein H., Lehmann M., Korge G. (1999). Structure and Regulation of the Salivary Gland Secretion Protein Gene Sgs-1 of Drosophila Melanogaster. Genetics.

[55] Garfinkel M.D., Pruitt R.E., Meyerowitz E.M. (1983). DNA Sequences, Gene Regulation and Modular Protein Evolution in the Drosophila 68C Glue Gene Cluster. J. Mol. Biol..

[56] Shore E.M., Guild G.M. (1986). Larval Salivary Gland Secretion Proteins in Drosophila Structural Analysis of the Sgs-5 Gene. J. Mol. Biol..

[57] Marin F., Luquet G., Marie B., Medakovic D. (2007). Molluscan Shell Proteins: Primary Structure, Origin, and Evolution. Current Topics in Developmental Biology.

[58] Lanio W., Swida U., Kress H. (1994). Molecular Cloning of the Drosophila Virilis Larval Glue Protein Gene Lgp-3 and Its Comparative Analysis with Other Drosophila Glue Protein Genes. Biochim. Biophys. Acta BBA—Gene Struct. Expr..

[59] Mateos B., Conrad-Billroth C., Schiavina M., Beier A., Kontaxis G., Konrat R., Felli I.C., Pierattelli R. (2020). The Ambivalent Role of Proline Residues in an Intrinsically Disordered Protein: From Disorder Promoters to Compaction Facilitators. J. Mol. Biol..

[60] Mettling C., Bourouis M., Richards G. (1985). Allelic Variation at the Nueleotide Level in Drosophilaglue Genes. Mol. Gen. Genet. MGG.

[61] Andres A.J., Fletcher J.C., Karim F.D., Thummel C.S. (1993). Molecular Analysis of the Initiation of Insect Metamorphosis: A Comparative Study of Drosophila Ecdysteroid-Regulated Transcription. Dev. Biol..

[62] Li T.-R., White K.P. (2003). Tissue-Specific Gene Expression and Ecdysone-Regulated Genomic Networks in Drosophila. Dev. Cell.

[63] Barnett S.W., Flynn K., Webster M.K., Beckendorf S.K. (1990). Noncoordinate Expression of Drosophila Glue Genes: Sgs-4 Is Expressed at Many Stages and in Two Different Tissues. Dev. Biol..

[64] Swida U., Lucka L., Kress H. (1990). Glue Protein Genes in Drosophila Virilis: Their Organization, Developmental Control of Transcription and Specific MRNA Degradation. Development.

[65] Kress H. (1981). Ecdysone-Induced Puffing InDrosophila: A Model. Naturwissenschaften.

[66] Kress H. (1982). Biochemical and Ontogenetic Aspects of Glycoprotein Synthesis in Drosophila Virilis Salivary Glands. Dev. Biol..

[67] Ramesh S.R., Kalisch W.-E. (1988). Glue Proteins InDrosophila Nasuta. Biochem. Genet..

[68] Ramesh S.R., Kalisch W.-E. (1989). Comparative Analysis of Glue Proteins in TheDrosophila Nasuta Subgroup. Biochem. Genet..

[69] Ramesh S.R., Shivanna N. (1999). SDS-PAGE Pattern Polymorphism of X-Chromosomal Glue Proteins in Natural Populations of Two Drosophila Nasuta Subgroup Species. Biochem. Genet..

[70] Perkowska E. (1963). Some Characteristics of the Salivary Gland Secretion of Drosophila Virilis. Exp. Cell Res..

[71] Thomopoulos G.N., Kastritsis C.D. (1979). A Comparative Ultrastructural Study of ‘Glue’ Production and Secretion of the Salivary Glands in Different Species of TheDrosophila Melanogaster Group. Wilhelm Rouxs Arch. Dev. Biol..

[72] Paris M., Boyer R., Jaenichen R., Wolf J., Karageorgi M., Green J., Cagnon M., Parinello H., Estoup A., Gautier M. (2020). Near-chromosome level genome assembly of the fruit pest Drosophila suzukii using long-read sequencing. Sci. Rep..

[73] Shirk P.D., Roberts P.A., Harn C.H. (1988). Synthesis and Secretion of Salivary Gland Proteins in Drosophila Gibberosa during Larval and Prepupal Development. Rouxs Arch. Dev. Biol..

[74] Hsu W.S. (1948). The Golgi Material and Mitochondria in the Salivary Glands of the Larva of Drosophila Melanogaster. J. Cell Sci..

[75] Gregg T.G., McCrate A., Reveal G., Hall S., Rypstra A.L. (1990). Insectivory and Social Digestion InDrosophila. Biochem. Genet..

[76] Ashburner M. (1970). Function and Structure of Polytene Chromosomes During Insect Development. Advances in Insect Physiology.

[77] Jones N.A., Kuo Y.M., Sun Y.H., Beckendorf S.K. (1998). The Drosophila Pax Gene Eye Gone Is Required for Embryonic Salivary Duct Development. Development.

[78] Beňová-Liszeková D., Mentelová L., Babišová K., Beňo M., Pechan T., Chase B.A., Farkaš R. (2021). An Apocrine Mechanism Delivers a Fully Immunocompetent Exocrine Secretion. Sci. Rep..

[79] Seyahooei M.A., Kraaijeveld-Smit F.J.L., Kraaijeveld K., Crooijmans J.B.M., Van Dooren T.J.M., Van Alphen J.J.M. (2009). Closely Related Parasitoids Induce Different Pupation and Foraging Responses in Drosophila Larvae. Oikos.

[80] Rossi G., Manfrin A., Lutolf M.P. (2018). Progress and Potential in Organoid Research. Nat. Rev. Genet..

[81] Low L.A., Mummery C., Berridge B.R., Austin C.P., Tagle D.A. (2021). Organs-on-Chips: Into the next Decade. Nat. Rev. Drug Discov..

